# Cost-effectiveness of integrative oncology for sustainable and patient-centred cancer care: a systematic review in the context of the WHO global traditional medicine strategy 2025–2034

**DOI:** 10.3389/fpubh.2026.1773455

**Published:** 2026-03-18

**Authors:** Friedemann Schad, Anja Thronicke, Thomas Reinhold, Shiao Li Oei, Hiba Boujnah, Elisa Baldini, Henrik Szőke, Nina Fuller-Shavel, Georg Seifert

**Affiliations:** 1 Research Institute Havelhöhe, Berlin, Germany; 2 Interdisciplinary Oncology and Palliative Care, Hospital Gemeinschaftskrankenhaus Havelhöhe, Berlin, Germany; 3 Charité Competence Center for Traditional and Integrative Medicine (CCCTIM), Charité – Universitätsmedizin Berlin, Berlin, Germany; 4 Charité - Universitätsmedizin Berlin, Institute for Social Medicine and Health Economics, Berlin, Germany; 5 EUROCAM, Brussels, Belgium; 6 International Federation of Anthroposophic Medical Associations (IVAA), Brussels, Belgium; 7 Synthesis Clinic, Berkshire, United Kingdom; 8 NCIO (National Centre for Integrative Oncology), Berkshire, United Kingdom

**Keywords:** cost-effectiveness, health policy, integrative oncology, TCIM, value based care, WHO

## Abstract

**Introduction:**

The World Health Organisation (WHO) recently released the Global Traditional Medicine Strategy 2025–2034, calling for strengthened evidence, governance, and integration of safe, effective traditional and complementary practices into national health systems. In oncology, integrative oncology (IO) combines evidence-informed complementary and traditional modalities with standard cancer care to improve symptom control, quality of life, and patient-centred outcomes. Although clinical benefits are increasingly recognised, comprehensive economic evidence remains limited, constraining reimbursement and policy implementation.

**Methods:**

This systematic review synthesised cost-effectiveness analyses of IO interventions and evaluated their implications for sustainable cancer care within the WHO Traditional, Complementary and Integrative Medicine (TCIM) framework. Searches of PubMed, EMBASE, PsycINFO, EconLit, and CINAHL identified peer-reviewed CEAs, cost-utility, or cost–benefit studies comparing IO with conventional oncology care.

**Results:**

Ten studies met inclusion criteria, comprising six randomised controlled trials, three real-world data analyses, and one model-based evaluation from Europe, Australia, Asia, and the United States. Interventions included psychological, phytotherapeutic, rehabilitative, and mind–body programmes. Across all modalities, IO interventions were either dominant or cost-effective within national willingness-to-pay thresholds. Methodological quality was high (mean CHEERS score 93%), although few studies addressed longterm outcomes, structural uncertainty, or equity impacts.

**Discussion:**

Overall, IO approaches showed favourable or dominant cost-effectiveness with phytotherapeutic and mind–body interventions revealing the most favourable cost-effectiveness profiles, followed by rehabilitative and psychological programmes economic results. As cost-effectiveness analyses are not yet available for all integrative oncology modalities, this systematic review represents an initial synthesis; the findings indicate a consistent trend towards cost-effectiveness, while generalisability across all IO approaches remains to be established. These findings align with the priorities of the WHO Global Traditional Medicine Strategy 2025–2034 and suggest that integrative oncology may support patient-centred, sustainable, and culturally sensitive cancer care, potentially supporting its value-based integration into national reimbursement frameworks.

**Systematic review registration:**

https://www.crd.york.ac.uk/PROSPERO/view/CRD420251019386, CRD4201019386.

## Highlights

- The WHO Global Traditional Medicine Strategy 2025–2034 identifies evidence as essential for integrating complementary and traditional practices in cancer care.
- This review synthesised 10 cost-effectiveness evaluations of integrative oncology as part of TCIM interventions across Europe, Asia, Australia, and the United States.
- Phytotherapeutic, rehabilitative, psychological, and mind–body integrative oncological approaches were consistently cost-effective or dominant compared with standard care.
- Findings indicate that integrative oncology as part of TCIM could be meaningfully incorporated into national cancer programmes and health insurance coverage.

## Introduction

Each year, more than 20 million people worldwide are newly diagnosed with cancer (1). This high incidence places a growing burden on patients, healthcare systems, and societies. In many countries, cancer is now among the leading causes of morbidity and mortality, challenging public health systems and calling for more comprehensive and sustainable approaches to care. Integrative oncology (IO) as part of Integrative Medicine is increasingly recognised in this development (2). It is defined as “a patient-centred, evidence-informed field of cancer care that uses mind–body practices, natural products, and lifestyle modifications alongside conventional treatments” (3). The integration of conventional medicine with Traditional, Complementary and Integrative Medicine (TCIM) is also a strategic priority of the WHO. In its Global Traditional Medicine Strategy 2025–2034, the WHO explicitly identifies research and evidence generation as further core pillars, alongside governance, service delivery, and workforce development (4). The strategy calls for strengthened clinical and health-services research to support the safe and effective integration of TCIM into national health systems. In parallel, the WHO aims to improve access to qualified TCIM practitioners, ensure the quality and availability of TCIM medicinal products, and develop reimbursement policies that respect patients’ rights to informed choice about their treatments (4).

Economic evaluations assessing the cost-effectiveness of IO as part of TCIM interventions are of particular importance in view of limited healthcare resources. However, this field is still underrepresented in research, making it difficult to assess long-term financial sustainability and feasibility. Previous systematic reviews are rare and either call for more rigorous studies (5, 6) or do not focus specifically on oncology (7). Earlier reviews also showed considerable methodological heterogeneity and limited reporting of incremental cost-effectiveness ratios (ICERs) or reimbursement aspects (5, 8, 9). Further work is needed to explore access to TCIM for underserved populations, to assess affordability across health systems, and to apply distributional cost-effectiveness methods (10). Given that the WHO Global Traditional Medicine Strategy 2025–2034 explicitly prioritises equity, access, and fairness, the current lack of distributional cost-effectiveness analyses represents a substantial evidence gap that future research should address. Such approaches would strengthen both the methodological and ethical dimensions of this research and better support equity-oriented decision-making in cancer care. This aligns with global efforts to advance more person-centred and equitable cancer care. However, despite increasing clinical and policy interest, no peer-reviewed systematic review has so far summarised the cost-effectiveness of IO across different modalities and perspectives, or explicitly aligned this synthesis with the WHO Strategy 2025–2034.

This *Oncology-Cost-Effectiveness Analysis of Integrative Concepts* (OCEANIC) systematic review therefore builds on previous work by providing the first synthesis framed around this WHO strategy. It compares diverse IO modalities and economic perspectives within one analytical framework (4). The review aims to (i) describe the methodological quality of existing IO cost-effectiveness studies, (ii) compare outcomes and cost-effectiveness ratios across IO interventions and settings, and (iii) identify research and policy gaps in relation to the WHO Global traditional medicine strategy’s goals on evidence generation and implementation. The findings are intended to inform policymakers, clinicians, and researchers about the value and feasibility of integrating TCIM approaches into routine oncology care. In line with the WHO TCIM pillars, the review considers governance, evidence generation, workforce development, service integration, and partnerships in the context of cost-effectiveness and value-based integrative cancer care (4). The review focuses on adult cancer populations in inpatient and outpatient oncology settings; paediatric or non-IO applications were not included.

## Methods

### Study description and protocol version

The systematic review was conducted in accordance with the PRISMA guidelines (7), see Supplementary Table S1. Record screening concluded on 30 June 2025, and data extraction on 31 July 2025. The current protocol version is 2.0, dated 4 December 2025. The review protocol was registered in the international prospective register of systematic reviews PROSPERO from the National Institute for Health and Care Research (CRD420251019386) (11). No major deviations occurred, except for post-hoc reconstruction of cost-effectiveness acceptability curves (CEACs) and normalisation of relative ICERs for cross-country comparison. The review was conducted in accordance with the Consolidated Health Economic Evaluation (CHEERS) Reporting Standards (12).

### Inclusion and exclusion criteria

Eligibility criteria were defined according to the Population, Intervention, Comparison, Outcome (PICO) framework (13). In line with the WHO Global traditional medicine strategy 2025–2034, which calls for strengthened evidence on safety and effectiveness, this review focused on TCIM interventions used in oncology, referred to here as integrative oncology (IO) therapies. Only studies published in peer-reviewed journals were considered. The systematic review encompassed both randomised controlled and non-randomised studies in accordance with its study protocol, the Cochrane Handbook for Systematic Reviews of Interventions and the CHEERS 2022 standards for economic evaluation reporting (11, 12, 14). Included studies reported data from adults diagnosed with a primary oncological disease and investigated IO therapy in this patient population. Each eligible study compared at least two groups, of which one received an IO intervention. The comparator consisted of at least one conventional, standard oncological, or guideline-oriented approach, which could also include ‘watch-and-wait’ strategies. Furthermore, only studies that assessed cost-effectiveness, cost–benefit, cost-utility, or the broader economic impact of IO interventions were retained. Each study was also required to specify at least one recognised analytical perspective—such as societal, third-party payer, hospital, or employer (11). No minimum follow-up duration was predefined; studies with horizons ranging from short-term (≤12 months) to long-term or life-time model projections were eligible. Preclinical studies were excluded. Likewise, studies lacking an economic evaluation component—as well as abstracts, conference proceedings, and other non-peer-reviewed sources—were not considered (11). In addition, studies focusing on TCIM without a direct link to cancer care were excluded from the review (11).

### Integrative oncology and traditional complementary and integrative medicine

Integrative oncology (IO) is defined as a patient-centred, evidence-informed field of cancer care that uses mind–body practices, natural products, and lifestyle modifications alongside conventional oncologic treatments to optimise outcomes and quality of life (3). Within the broader framework of Traditional, Complementary and Integrative Medicine (TCIM), IO represents an oncology-specific application of a holistic care model that combines conventional medicine with complementary practices and traditional medical systems to address physical, psychological, and social dimensions of health (15). To ensure conceptual transparency, we classified certain conventional supportive care interventions, such as standard psycho-oncological psychotherapy, within the IO framework when delivered as part of coordinated, patient-centred supportive care alongside anticancer treatment. These interventions represent core mind–body and psychosocial components of IO; their inclusion was therefore based on functional contribution to integrative, multimodal supportive care rather than disciplinary origin, consistent with contemporary IO and TCIM frameworks (4, 15).

### Information sources and search strategy

A comprehensive and sensitive topic-based search was performed for each database from inception to 7 April 2025, which served as the predefined cut-off date for study inclusion, without language or geographical restrictions. The databases included PubMed, EconLit, EMBASE, PsycInf, and Cumulative Index to Nursing and Allied Health Literature. The strategy incorporated Boolean search strings combining key terms related to IO, health economic evaluation, and oncology treatment models, see search strategy and terms in Supplementary Table S2. For all economic data, costs were extracted in the original study currency and with the cost year as reported in each publication. When available, we documented whether authors had adjusted costs for inflation or purchasing-power parity relative to their stated reference year. No additional inflation or purchasing power parity adjustments were applied by the reviewers. For studies lacking explicit cost-year information, the publication submission year was used as a proxy. Comparability across studies was ensured by normalising ICERs to national willingness-to-pay (WTP) thresholds rather than converting currencies. Additional studies were identified by contacting authors and experts, screening the reference lists of included reviews, and searching relevant trial and study registries.

### Data items, management, selection and collection process

Two independent reviewers systematically searched all information sources and screened titles and abstracts, grading each as eligible, not eligible, or might be eligible (16, 17). Study records were managed in EndNote, a dedicated reference management software (18). Records that could not be clearly excluded from title and abstract alone were retained for discussion (13). Both reviewers then assessed the full texts against predefined criteria, documenting reasons for exclusion, and checked for completeness and consistency. Discrepancies were resolved by discussion, and if needed, a third reviewer provided the final decision (10). When required, study authors were contacted to supplement missing information. A standardised form captured study design, year of publication, country, sample size, cancer type, type of IO care, type of economic evaluation (cost-effectiveness, cost-utility, or cost–benefit), cost perspective, and key outcomes, including timeframes.

### Outcomes, risk of bias and study-level quality assessment

Key economic measures—ICERs, cost–benefit ratios, and related incremental metrics—were used to assess the overall value of interventions. When costs and outcomes were expressed in comparable units (e.g., cost per QALYs gained or cost per life-year gained) they were compared separately to ensure consistent interpretation across studies. Relative ICERs were calculated by dividing each study’s reported ICER by the respective country-specific WTP benchmark—Germany €50,000 per life year gained (LYG); Australia AU$50,000 per QALY; UK £30,000 per QALY; Taiwan NT$1,792,062 per QALY; USA US$ 50,000 per QALY. The benchmark of US $50,000 per QALY for cost-effectiveness, originally introduced in US health economics, continues to serve as a pragmatic international reference despite ongoing debate about its validity and evolution (19). This approach does not constitute a formal economic pooling and was performed to facilitate comparability with European cost-effectiveness literature; assumption is not intended to be interpreted as a normative threshold for reimbursement or policy decisions but serves as an analytical benchmark only. Germany does not have an officially defined threshold for reimbursement, however model-based analyses suggest plausible ranges between €42,600 and €88,100 per LYG (20). Therefore, €50,000 per LYG was applied as a pragmatic analytical benchmark. In the UK, the National Institute for Health and Care Excellence has announced plans to revise and increase its current £30,000 per QALY threshold range in spring 2026 to better reflect updated economic conditions. However, as this review was intended to be submitted in December 2025, the threshold of £30,000 was kept (21). Comparative analyses evaluated both costs and outcomes, including clinical and patient-reported effects. Studies reporting non-utility outcomes (e.g., cost per unit change in distress or per unit change in energy) were retained for completeness but were not converted into cost per QALY estimates, as no validated mapping functions exist between these instruments and standard health-utility scales (22). Such results were synthesised descriptively to avoid artificial precision or bias. CEACs were derived from published probabilistic sensitivity analysis (PSA) data, when available, using logistic interpolation across willingness-to-pay thresholds. To ensure comparability across currencies and health-system contexts, all CEACs were normalised to a relative WTP scale. All probabilistic and decision-modelling approaches followed established health economic methods (23, 24).

Methodological and reporting quality were evaluated using the CHEERS 2022 checklist (12). Certainty of evidence was not formally graded, as the heterogeneity of study designs and outcomes precluded a standardised GRADE assessment. Disagreements were resolved by consensus, and risk of bias for each included study due to missing results was additionally examined and independently assessed by both reviewers. When essential data were unclear or unavailable, study authors were contacted for clarification. Each CHEERS item was scored on a 0–2 scale (0 = not reported; 1 = partly reported; 2 = fully reported). Scores were aggregated into four domains (i) reporting and transparency, (ii) costing methods, (iii) patient and stakeholder engagement, and (iv) sensitivity and uncertainty analysis (12) and adapted methodological quality approaches used in recent systematic reviews of health economic evaluations (25, 26). For each domain, percentage scores were calculated as domain score % = (sum of item scores ÷ maximum possible score) × 100. Domain percentages were averaged across studies to obtain comparative performance profiles. A global methodological quality score was additionally computed by summing all CHEERS item scores per study and expressing the result as a percentage of the maximum attainable score, providing an overall benchmark of reporting completeness. All descriptive and quantitative analyses, including domain-percentage plots and CEAC reconstructions, were conducted in R version 4.3.2 (R Foundation for Statistical Computing, Vienna, Austria) using ggplot2, and BCEA packages to support visualisation, and economic modelling.

### Data synthesis and confidence in cumulative evidence

Owing to the heterogeneity of economic evaluations, a meta-analysis was not undertaken. Heterogeneity arose from differing study designs, cost perspectives, outcome units, time horizons, currencies, and national thresholds. Instead, a structured narrative synthesis assessed study quality, methodological differences, and overall robustness. Data extraction focused on methodological characteristics, intervention strategies, and key economic outcomes. Studies were systematically classified according to design type and intervention strategy, while their core elements were organised using the PICO framework, see eligibility criteria. Summarised datasets were presented in structured evidence tables. Subgroup analyses were performed where sufficient data permitted.

## Results

### Study selection

**Figure 1:**
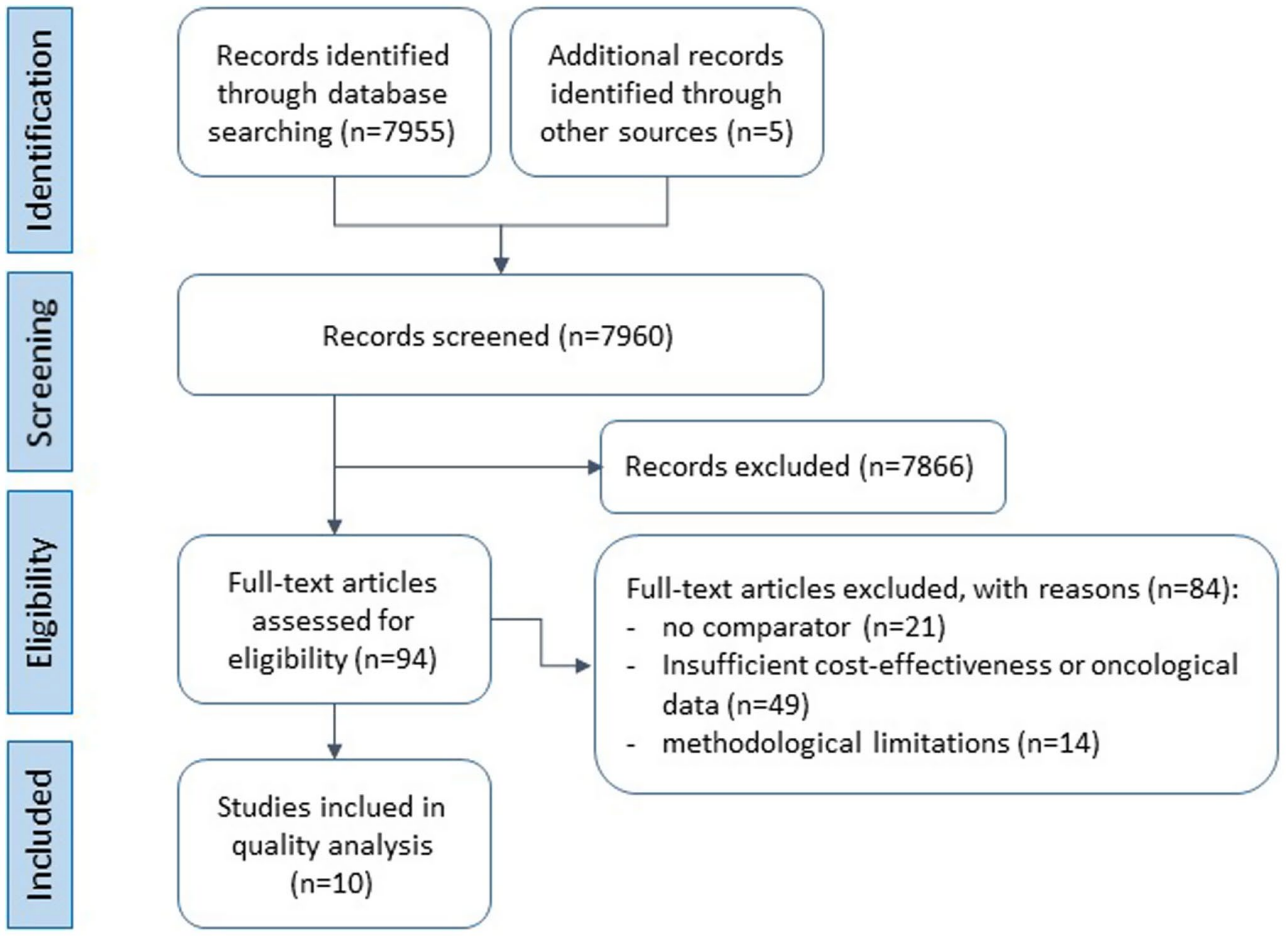
PRISMA flow diagram of study selection. Flow of records through the screening and selection process for CEAs of IO interventions (studies included in the final synthesis, *n* = 10).

A total of 7,955 records were identified through database searches and five additional records through other sources, yielding 7,960 unique records, see Figure 1. After title and abstract screening, 7,866 publications were excluded for not meeting predefined eligibility criteria. The majority (*n* = 7,441) did not evaluate IO interventions. Further exclusions comprised reviews (*n* = 359), study protocols (*n* = 42), and other non-eligible reports (*n* = 24), including studies without a comparator (*n* = 5), those not reporting cost-effectiveness outcomes (*n* = 9), and those lacking an identifiable economic perspective or relevant data (*n* = 10). The remaining 94 full-text articles were assessed for eligibility. 84 full-text articles were excluded with 49 lacking real cost-effectiveness data or oncology-relevance, 21 having no comparator, and 14 not meeting minimum methodological quality criteria. Ultimately, 10 studies fulfilled all inclusion criteria and were retained for the final qualitative synthesis, see Figure 1.

### Characteristics of included studies

The 10 included CEA studies were conducted between 2008 and 2024 and spanned diverse health systems across Europe (Germany, the United Kingdom, France), Australia, Taiwan, and the United States (Table 1) (13, 16, 27–34). Study designs included randomised controlled trials (RCTs, *n* = 6) (29–34), real-world data studies (RWD, *n* = 3) (13, 16, 27), and model-based simulations (*n* = 1) (28). Together, these studies represent an evolving methodological spectrum ranging from trial-based economic evaluations to large RWD analyses (Table 1).

**Table 1:** Characteristics of included studies.

Author, year, country	Study type	Cancer type, stage (P)	Sample size (P)	Type of integrative oncology (I)	Comparator (C)	Type of economic evaluation	Time horizon, discount rate, cost year, currency	Economic perspective	Main outcomes (O)
Mandelblatt, 2008, U. S. (34)	RCT, 3 arm	Invasive breast cancer 4–6 weeks after surgery without neoadjuvant or high-dose chemotherapy	389	Add-on video psychoeduction with or without counselling^3^	Usual Care: booklet psychoeduction “Moving beyond cancer” only	CEA	6 and 12 months, not applied due to short follow-up time (≤1 year), 2002, US$	Societal	ICER (expressed in cost-per-point-of-a-PROM)
Molassiotis, 2013, UK (31)	RCT, 3-arm, sham controlled	Mixed cancer	238	Acupressure	Conventional antiemetic medication with or without sham or real wristband	CUA	Up to 4 cycles chemotherapy, no discount, 2010, GBP	Healthcare service provider, societal	ICER (cost per QALY gained)
Mourgues, 2014, France (29)	RCT, tricentric	Working, inactive or retired breast cancer patients in complete remission	181	Multimodal add-on thermal treatment with physical activity & dietary consultation^4^	Usual care, dietitian consultation only	CEA	12 months, 0,3,5%, 2008–2010, EUR	Societal	Descriptive
Round, 2014, UK (30)	RCT, single centre	Progressive recurrent breast and haematological cancer	41	Standardised add-on complex hospice based day therapy rehabilitation intervention^2^	Usual Care, wait-list control	CUA	3 months, no discount rate 2010–2011, GBP	Public healthcare and social care payer perspective	ICER (cost per QALY gained)
Shih, 2019, Australia (33)	RCT	Breast cancer colorectal cancer and melanoma	109	Psychological support	Usual care: no structured psychological	CUA	6 months to 15 months, no discount, 2013, AU$	Healthcare sector	ICER (cost per QALY gained)
Gordon, 2024, Australia (32)	RCT	Benign and malignant brain tumour	82, model: 100	Telehealth supportive psychological support intervention^1^	Conventional care	CUA	6 months, adjustments to inflation where needed, 2022, AU$	Healthcare sector	Incremental QALY gain, probabilistic decision metric
Dholaka, 2021, U. S. (28)	Model-based	Epithelial ovarian cancer; medically frail women undergoing PDS for EOC in 1 year	4,415 (hypothetical cohort)	Multimodal intervention including physical, nutritional, psychological counseling	Usual care: primary surgical intervention	CEA	Life-long, not reported, 2018–2019, US$	Healthcare sector	ICER (cost per LYG)
Tang, 2024, Taiwan (27)	RWD	Non-small cell lung cancer	43,122	Add-on Chinese herbal medicine	Conventional care: non-TCM user	CEA	5-years post-diagnosis, 3%, 2000–2018, NT$	Healthcare sector	ICER (cost per LYG)
Thronicke, 2020a, Germany (13)	RWD, registry-based, monocentric	Stage IV pancreatic cancer	88	Add-on Viscum album L.	Conventional care: Chemotherapy	CEA	24 months from diagnosis, no discount rate, 2012–2017, EUR	Hospital/ provider	ICER (cost per LYG)
Thronicke, 2020b, Germany (16)	RWD, registry based, monocentric	Stage IV non-small cell lung cancer	118	Add-on Viscum album L.	Conventional care: Chemotherapy	CEA	12–24 months, no discount rate, 2012–2016, EUR	Hospital/ provider	ICER (cost per LYG)

Characteristics of included studies. PDS, primary debulking surgery; EOC, epithelial ovarian cancer; RWD, real-world data study; CEA, cost-effectiveness, CUA, cost-utility analysis; HCP, health-care provider, LYG, life-years gained; QALY, quality-adjusted life year, ICER, incremental cost-effectiveness ratio.

1 10 × 1-h weekly sessions of add-on psychological support by a psychologist using zoom videoconferencing.

2 Add-on complex outpatient day therapy rehabilitation intervention including physical and psychological therapies.

3 Add-on video psychoeducation “Moving beyond cancer”.

4 2-week 2-h multicomponent add-on cancer rehabilitation programme comprising spa treatment with dietary physical education, running and physiotherapy.

Across tumourr entities, CEA investigations encompassed breast (29, 30, 33, 34), lung (16, 27), pancreatic (13), colorectal (33), ovarian (28), melanoma (33), brain (32) or mixed cancer populations (31, 33), mainly in advanced or survivorship stages. The types of IO interventions varied from add-on *Viscum album L.* therapy (13, 16) and traditional Chinese herbal medicine (27) to psychological support and counselling (28, 32–34), acupressure (31), and multimodal rehabilitation programmes (28, 29), each compared with conventional oncology care or standard supportive-care measures. Most analyses were undertaken from a healthcare provider (27–33) or hospital perspective (13, 16, 29), while several also incorporated societal perspective (31, 34). Time horizons ranged from short-term (3–12 months) in RCTs (29–34) over long-term (2 to 5 years post-diagnosis) in RWD (13, 16, 27) to life-long measures in model-based studies (28). Economic outcomes were primarily reported as ICERs which were expressed as LYG (13, 16, 27, 28) or cost per QALY gained (30–33), except in cases where the intervention was dominant. One study reported the cost–benefit ratio as cost per unit of patient-reported outcome improvement (34).

### Main cost-effectiveness outcomes

Across the 10 included studies, favourable or dominant cost-effectiveness profiles for IO interventions compared with conventional care were shown, see Table 2. In all studies calculated cost-effectiveness ratios ranged from dominant, cost-saving to modest additional costs per LYG or per QALY gained indicated by an ICER below their respective national WTP thresholds, see Table 2. Among the six randomised controlled trials, all three psychosocial interventions were cost-effective or cost-neutral (32–34), see Table 2. Gordon et al.’s randomised telehealth psychological-support intervention achieved an incremental gain of 0.03 QALYs and an 87% probability of cost-effectiveness at an Australian threshold of AU$50,000 per QALY indicating high cost-effectiveness of this intervention (32). Mandelblatt et al. showed that an add-on psycho-educational video intervention was clinically and economically favourable compared with a booklet psycho-education only at US$ 7,275 per one-point improvement in distress and US$ 2.22 per unit energy improvement (34). Shih et al. reported an ICER of AU$34,300 per QALY gained for a psychological-support programme with a 53% probability of cost-effectiveness at the AU$50,000 per QALY threshold (33). Designed as a mind–body RCT, Molassiotis et al. found that acupressure dominated standard antiemetic care but was slightly less effective and less costly than sham acupressure converting to an ICER of £7,359.68 per QALY gained (31). Three rehabilitative interventions (two RCTs and one model-based) were either cost-effective or dominant, see Table 2. Round et al. identified a 0.052 QALY gain for a hospice-based day-therapy rehabilitation programme, yielding an ICER of £14,231 per QALY gained and a 45.2% probability of cost-effectiveness in bootstrap sensitivity analyses at the £30,000 per QALY threshold at 6 months (30). An IO rehabilitation programme by Mourgues et al., comprising spa-based physical activity, dietary consultation and physiotherapy, compared with standard dietetic counselling alone, were less costly with similar or improved outcomes, revealing dominance (29). The third rehabilitative study, a model-based evaluation by Dholiaka et al. on a multimodal supportive-care programme was found to be dominant as well (28). Here, the simulation in medically frail women with epithelial ovarian cancer remained cost-effective at up to US$9,400 per patient versus usual surgical care, from a healthcare payer perspective (28). Phytotherapeutic therapies reinforced findings of cost-effectiveness of IO in long-term observed cohorts (13, 16, 27). Both publications from Thronicke et al. revealed cost-effectiveness of add-on *Viscum album* L. therapy in advanced or metastasized pancreatic cancer and non-small cell lung cancer (NSCLC), with ICERs of €7,539 and €3,586 per LYG, respectively, well below a national average WTP of €50,000 per LYG, from hospital’s perspective (13, 16). Tang et al. reported that add-on phytotherapeutic Chinese medicine applied in NSCLC patients yielded an ICER of NT$880,908 per LYG with a greater than 90% probability of being cost-effective at the nationally applied WTP, indicating improved survival at acceptable cost from the Taiwanese payer perspective based on five-year insurance claim data (27), see Table 2.

**Table 2:** Summary of main cost-effectiveness outcomes.

Categories	Mandelblatt, RCT (34)	Molassotis, RCT (31)	Mourgues, RCT (29)	Round, RCT (30)	Shih, RCT (33)	Gordon, RCT (32)	Dholiaka, model-based (28)	Tang, RWD (27)	Thronicke, a, RWD (13)	Thronicke, b, RWD (16)
Intervention category	psychological	mind–body	rehabilitative	rehabilitative	psychological	psychological	rehabilitative	phytotherapy	phytotherapy	phytotherapy
ICER or cost–benefit ratio	US$7,275 per unit distress improvement US$2.22 per unit energy improvement	Slightly less effective than sham £7,359.68/QALY gained	slightly higher costs but greater effectiveness: judged cost-efficient^12^	£14,231/QALY gained	AU$ 34,300/QALY gained	dominant	dominant	NT$ 880.908/LYG	7,539.32€ per LYG	3,585.84€ per LYG
% of likelihood of being cost-effective (at WTP)	NA (no PSA conducted)	70% probability at £20,000 per QALY compared to standard	NA (no PSA conducted)	9.5% at £30,000 per QALY (3 m); 45.2% at £30,000 per QALY (6 m)	53% at AU$ 50,000 per QALY	87% at AU$ 50,000 per QALY	NA (no PSA conducted)	Bootstrap: all simulations below 3 × GDP per-capita threshold, indicating very high probability of cost-effectiveness	bootstrap: majority (ca. 88%) of replications in the northeast quadrant, indicating high probability of cost-effectiveness	bootstrap: appr. 74% of replications in the northeast quadrant, supporting cost-effectiveness
Incremental QALYs^1^	NA	−0.012	NA	0.052	0.0142	0.03	NA	NA	NA	NA
Main finding	cost-effective	cost-effective at low willingness-to-pay thresholds	dominant	cost-effective (6 m)	cost-effective	dominant	dominant	cost-effective	cost-effective	cost-effective
Limitations	educated participants with income bias, no QALY/LYG outcomes, efficacy measured only in symptom units	short horizon	no QALY/LYG outcomes, efficacy measured only in hours of activity	3-month horizon, single centre, small sample size	missing data in resource use, short horizon	Covid-19 limited returning signed paper forms, reduced access to services, imputation of data	model-based, hypothetical cohort	RWD, residual confounding	RWD, single centre, transferability	single-centre RWD

Summary of main cost-effectiveness outcomes. LYG, life-year gained; QALY, quality-adjusted life year; PROM, patient-reported outcome measure; GDP, gross domestic product; TCM, Traditional Chinese Medicine; WTP, willingness-to-pay; LOS, length of stay; cost–benefit-ratio: cost per life-year gained (LYG), cost per QALY gained, or cost per point improvement in a patient-reported outcome measure (PROM).

1 NA; incremental QALYs were not reported or could not be derived from the published data.

### CHEERS-based assessment of reporting quality and risk of bias

Overall reporting scores ranged from 84 to 100% within IO groups with a mean of 93%, indicating strong adherence to CHEERS standards (12). Core elements, such as study background, objectives, population, comparators, perspectives, and main results, were consistently well described. However, several methodological limitations were identified. Justification of discount rates and reporting of currency conversions or price-year adjustments were incomplete in several studies. A pre-specified health-economic analysis plan was fully reported in one study (30), and partly reported in four (28, 31, 33, 34), and absent in five (13, 16, 27, 29, 32).

**Figure 2:**
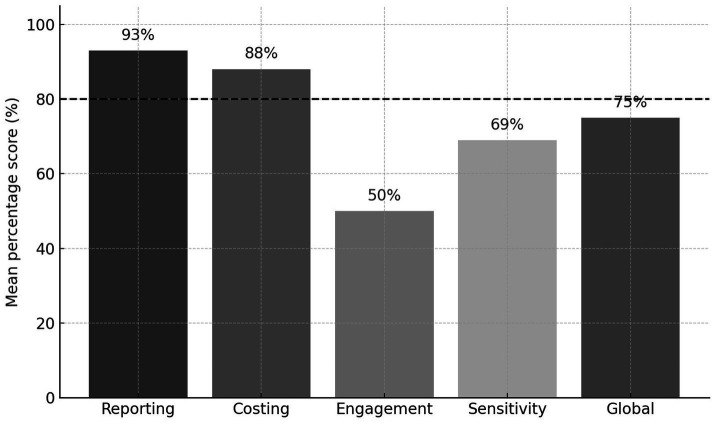
CHEERS-based quality assessment across methodological domains including global score. Mean percentage scores (0–100%) across the four domains (reporting, costing, engagement, sensitivity) and global score among 10 included cost-effectiveness analyses. Scores were calculated as the proportion of maximum achievable points. The dashed line indicates the 80% threshold for good reporting quality. Scores are shown as percentage of maximum achievable points per domain (0–2 scale per item); CHEERS, Consolidated Health Economic Evaluation Reporting Standards.

Costing approaches were generally transparent but heterogeneous in scope and valuation. With mean scores within IO groups ranging from 83 and 100% and a global mean of 88%, the costing domain reflected high but not fully comprehensive adherence to CHEERs standards. Most studies clearly identified direct medical costs, although indirect or productivity-related costs were often omitted. Price-year and inflation adjustments lacked inconsistency, and unit-cost sources were rarely detailed. Stakeholder and patient involvement were inconsistently reported with a mean of 50%, see Table 3 and Figure 2. Only a minority of studies—Dholakia et al., Molassiotis et al., Round et al., and Shih et al. (28, 30, 31, 33)—explicitly mentioned engagement activities, mainly during intervention development, endpoint selection, or interpretation of results. The remaining studies provided minimal or no information on participatory processes, indicating that stakeholder input remains an underrepresented dimension of IO economic evaluations.

**Table 3:** Study quality according to CHEERS checklist items.

CHEERS categories	Mandelblatt RCT psych	Molassotis RCT MB	Mourgues RCT reha	Round RCT reha	Shih RCT psych	Gordon RCT psych	Dholakia model reha	Tang RWD phyt	Thronicke,a RWD phyt	Thronicke, b RWD phyt
Reporting										
Title and Abstract	2	2	2	2	2	2	2	2	2	2
Background and Objectives	2	2	2	2	2	2	2	2	2	2
Health Economic Analysis Plan	1	1	0	2	1	0	1	0	0	0
Study Population (R)	2	2	2	2	2	2	2	2	2	2
Setting and Location	2	2	2	2	2	2	2	2	2	2
Comparators	2	2	2	2	2	2	2	2	2	2
Perspective	2	2	2	2	2	2	2	2	2	2
Time Horizon	2	2	2	2	0	2	2	2	2	2
Choice of Health Outcomes	2	2	1	2	2	2	2	2	2	2
Measurement of Effectiveness	2	2	2	2	2	2	2	2	2	2
Measurement and Valuation of Preference-Based Outcomes	2	2	0	2	1	2	2	2	1	1
Study Parameters	2	2	2	2	2	2	2	2	2	2
Summary of main results	2	2	2	2	2	2	2	2	2	2
Study Findings, Limitations, Generalisability, and Current Knowledge	2	2	2	2	2	2	2	2	2	2
Source of Funding	2	2	2	2	2	2	2	2	2	2
Conflict of Interest	2	2	2	2	2	2	2	2	2	2
Points out of 32 points based on 16 items each scores 0–2	**31**	**31**	**27**	**32**	**28**	**30**	**31**	**30**	**29**	**29**
Percentage	**97**	**97**	**84**	**100**	**88**	**94**	**97**	**94**	**91**	**91**
Costing method										
Measurement and valuation of resources and costs	2	2	1	2	2	2	2	2	2	2
Currency, Price Date, and Conversion	1	2	1	2	2	2	2	2	1	1
Points out of 4 points based on 2 items each scores 0–2	**3**	**4**	**2**	**4**	**4**	**4**	**4**	**4**	**3**	**3**
Percentage	**75**	**100**	**50**	**100**	**100**	**100**	**100**	**100**	**75**	**75**
Exploratory methodological dimension										
Approach to Engagement with patients and others affected by the study	0	2	1	1	1	1	1	0	1	1
Effect of Engagement with patients and others affected by the study	1	1	0	2	2	1	2	0	1	1
Points out of 4 points based on 2 items each scores 0–2	**1**	**3**	**1**	**3**	**3**	**2**	**3**	**0**	**2**	**2**
Percentage	**25**	**75**	**25**	**75**	**75**	**50**	**75**	**0**	**50**	**50**
Sensitivity analyses										
Discount Rate	2	0	2	2	0	2	1	2	0	0
Rationale and Description of Model	2	2	0	2	2	2	2	2	2	2
Analytics and Assumption	2	2	1	2	2	2	2	2	2	2
Characterising Heterogeneity	2	2	1	1	2	1	2	2	1	1
Characterising distributional effects	0	0	0	0	0	0	0	0	0	0
Characterising Uncertainty	1	2	0	2	2	1	2	2	2	2
Effect of uncertainty	2	2	0	2	2	1	2	2	2	2
Points out of 14 points based on 7 items each scores 0–2	**11**	**10**	**4**	**11**	**10**	**9**	**11**	**12**	**9**	**9**
Percentage	**79**	**71**	**29**	**79**	**71**	**64**	**79**	**86**	**64**	**64**

RCT, randomised controlled trial; model, model-based; RWD, real-world data study; psych, psychological therapy; MB, mind–body therapy; reha, rehabilitative therapy; phyt, phytotherapy.

Bold, points and percentages per CHEERS subscore.

Several studies lacked comprehensive sensitivity analyses yielding a mean score of 69%, see Table 3 and Figure 2. Model-based and most RCT-based evaluations conducted probabilistic sensitivity analyses and presented CEACs (28–34), whereas RWD studies relied primarily on bootstrap resampling to quantify parameter uncertainty (13, 16, 27). Structural and scenario uncertainty were rarely explored. None of the included analyses explicitly assessed distributional or equity effects, and only a subset reported heterogeneity analyses through subgroup or interaction testing. These were conducted in Dholakia et al., Molassiotis et al., Tang et al., Mandelblatt et al., and Mourgues et al., typically examining variation by diagnosis, risk profile, or patient-reported outcomes (27–29, 31, 34). The aggregated global methodological quality score, derived from the mean domain-specific CHEERS scores, was 75%, indicating a generally modest level of completeness tempered by lower engagement scores despite strong performance in costing and reporting domains across IO modalities, see Figure 2.

### Comparative CHEERS performance by IO modality

When comparing the five CHEERS domains across IO modalities, several differences in quality were observed. Reporting quality was consistently high, with mean scores ranging from 92% (phytotherapy) to 97% (mind–body). Costing methods also performed well, particularly in mind–body interventions (100%), while rehabilitation and phytotherapy studies showed slightly lower completeness (both 83%). Stakeholder engagement reporting showed the greatest variability, here the mind–body study achieved the highest score (75%), whereas phytotherapy studies scored lower (33%). As all phytotherapy studies were based on RWD, patient involvement occurred mainly through routine clinical documentation rather than active participation in study design. Because CHEERS places greater weight on formal, study-targeted involvement, e.g., contributing to protocol development, these forms of participation are not fully recognised in the scoring system, resulting in lower engagement scores for RWD studies. Sensitivity and uncertainty analyses showed intermediate quality overall, ranging from 62% (rehabilitation) to 71% (psychological and phytotherapy).

## Diagnosis based subgrouping

In breast cancer (*n* = 4) psycho-oncological and rehabilitative programmes were uniformly cost-effective or cost-neutral, yielding improvements in health-related quality of life (HRQL) and psychosocial outcomes (29, 30, 33, 34), see Supplementary Table S3. Reported ICERs ranged from £14,231 per QALY gained (approximately €16,510 per QALY gained) to AU$34,300 per QALY gained (approximating to €20,600 per QALY gained), while others reported US$7,275 per PROM-point improvement (29, 30, 33, 34). In advanced lung cancer (*n* = 2), phytotherapeutic therapies achieved cost-effectiveness well below national thresholds, between €3,586 per LYG (*Viscum album* L.) and NT$880,908 (approximately €25,700 per LYG, traditional Chinese Medicine) with improved or comparable survival outcomes (16, 27). In advanced pancreatic cancer (*n* = 1), the phytotherapeutic intervention with *Viscum album L.* achieved cost-effectiveness well below the national threshold, with an ICER of €7,539 per LYG. In ovarian cancer (*n* = 1), the model-based simulation found a multimodal rehabilitation strategy to be dominant for patients with ovarian cancer, achieving lower costs and longer life expectancy than standard surgical care (28). Likewise, in brain tumour patients (*n* = 1), telehealth psychological support was dominant, with an 87% probability of cost-effectiveness at a WTP threshold of AU$50,000 per QALY (32). For mixed or multiple cancer populations (*n* = 2), mind–body and psychological support interventions showed cost-effectiveness within national thresholds, with incremental QALY gains ranging from 0.012 to 0.014 (31, 33).

### Economic based subgrouping

When stratified by economic perspective, cost-effectiveness conclusions remained consistent, see Supplementary Table S4. Studies adopting a hospital or provider perspective (*n* = 3) consistently demonstrated institutional cost savings (13, 16, 29). Under a healthcare payer perspective (*n* = 5), both RCTs and model-based studies reported ICERs well below national thresholds across currencies, with probabilistic analyses indicating 45–87% likelihood of cost-effectiveness, varying by comparator and analytic horizon. Reported studies were either cost-effective or dominant (27, 28, 30–32). Analyses conducted from a societal perspective (*n* = 2) also produced favourable results with estimates of US$ 7,275 per PROM point improvement and AU$34,300 per QALY gained (33, 34). The single mixed-perspective study found cost-effectiveness consistent across health-care, social-care, and societal perspective within the UK healthcare setting (31). The convergence of findings across these perspectives underscores that IO interventions provide measurable economic value at both institutional and system-wide levels.

### Intervention-based subgrouping

When grouped by intervention type, findings remained consistent across study designs and national contexts, see Supplementary Table S5. Phytotherapeutic interventions (*n* = 3) showed strong cost-effectiveness, with ICERs of €3,586 and €7,539 per LYG in German RWD studies (13, 16) and NT$880,908 per LYG in a Taiwan RWD study (27), the latter aligning with cost-effectiveness below a 3 times GDP threshold. Psychological programmes (*n* = 3) were cost-effective or dominant (32–34). Rehabilitative programmes (*n* = 3) likewise showed economic favourability, Dholakia and Mourgues et al. were dominant compared to standard, and Round et al. reported a 45.2% probability of cost-effectiveness (6-month horizon) at £30,000 per QALY (28–30). Mind–body therapies (*n* = 1) had the potential to be cost-effective at £7,359.68 per QALY gained versus sham, with a 70% probability at £20,000 per QALY compared to standard care (31).

**Figure 3:**
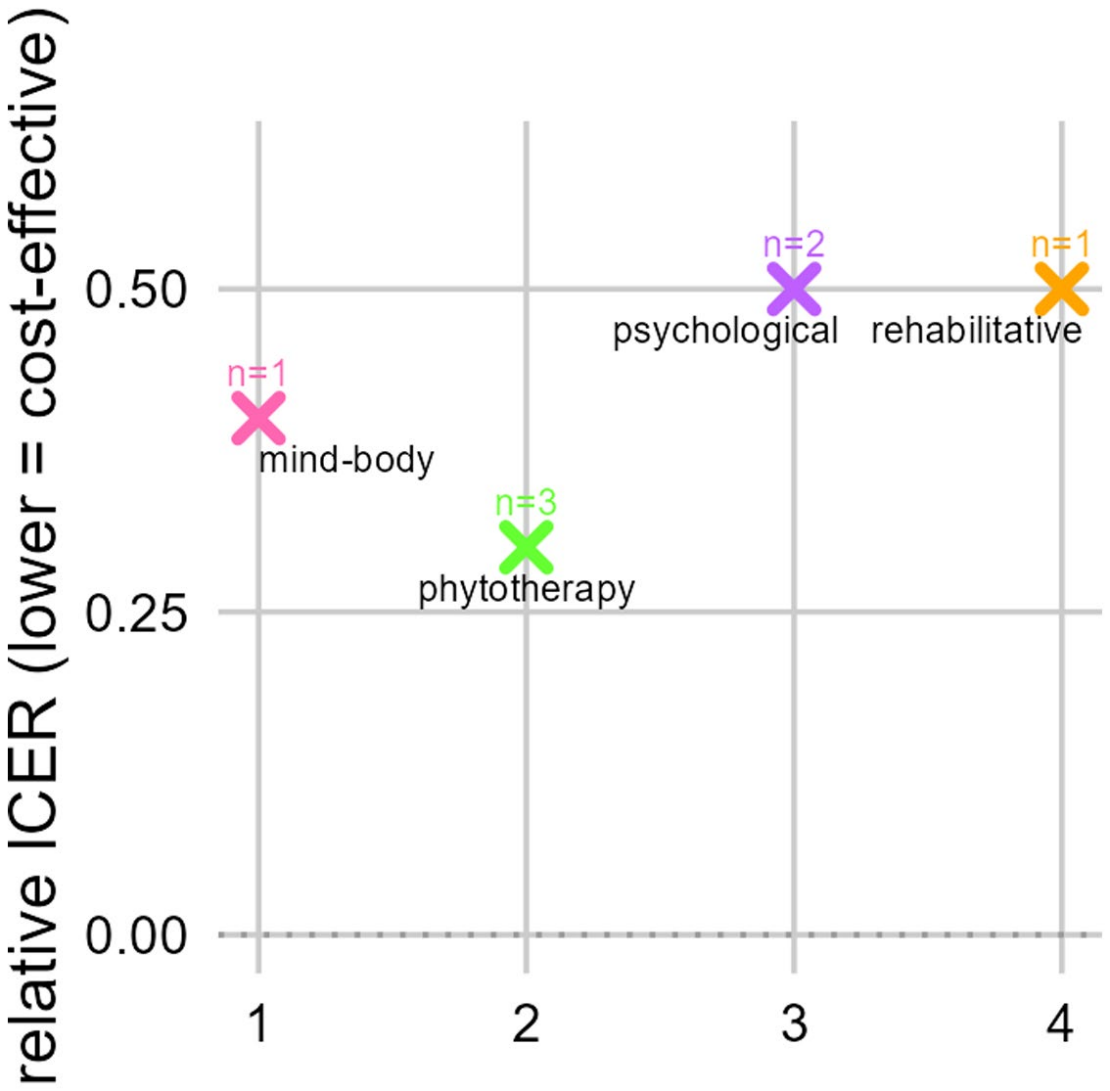
Subgroup analysis by intervention type showing relative cost-effectiveness across IO modalities. Each cross represents the standardised ICER for one intervention category. Lower relative ICER values indicate greater cost-effectiveness. Only studies reporting explicit ICER estimates were included in the subgroup analysis; dominant interventions and studies without classical ICER calculations were excluded. IO, integrative oncology; ICER, incremental cost-effectiveness ratio; pink, mind–body (Molassiotis); green, phytotherapy (Thronicke, Tang, Thronicke); purple, psychological (Mandelblatt; Shih); orange, rehabilitative (Round). Relative ICERs were normalised to study-specific willingness-to pay thresholds to allow exploratory cross-study comparison and should be interpreted cautiously given methodological heterogeneity and the small number of studies per category.

Figure 3 illustrates the mean relative ICER values across IO interventions categories based on studies reporting explicit ICER estimates (see also Supplementary Table S6). These values were normalised to study-specific willingness-to-pay thresholds to allow an exploratory, descriptive comparison across heterogeneous studies. Phytotherapeutic interventions (*n* = 3) including *Viscum album* L. and traditional Chinese herbal medicine, showed the lowest relative ICER values, with relative ICER values around 0.3 (13, 16, 27). Mind–body approaches (*n* = 1), such as acupressure, demonstrated a relative ICER value of approximately 0.4, indicating favourable cost-effectiveness as well (31). Rehabilitative programmes (*n* = 1) and psychological interventions (*n* = 2) each yielded relative ICER values of approximately 0.5, reflecting moderate but still cost-effective programmes (30, 33, 34).

### Cost-effectiveness uncertainty and probabilistic findings

Across the included cost-effectiveness analyses, uncertainty was addressed through a range of analytical approaches. Model-based and trial-based studies (28, 30–33) conducted PSA and presented CEACs, whereas real-world data studies applied bootstrap resampling to derive 95% confidence intervals for incremental costs and effects (13, 16, 27). One RCT lacked a formal uncertainty analysis (29). Three studies reporting probabilistic sensitivity analyses showed probabilities of cost-effectiveness ranging from approximately 45 to 87% across the evaluated interventions (30, 32, 33). These studies exhibited distinct CEA curve (CEAC) profiles. Round et al., representing a rehabilitative IO intervention, demonstrated the highest probability of cost-effectiveness, exceeding 50% at relatively low WTP thresholds and approaching values above 90%, see orange curve in Supplementary Figure 1 (30).

**Figure 4:**
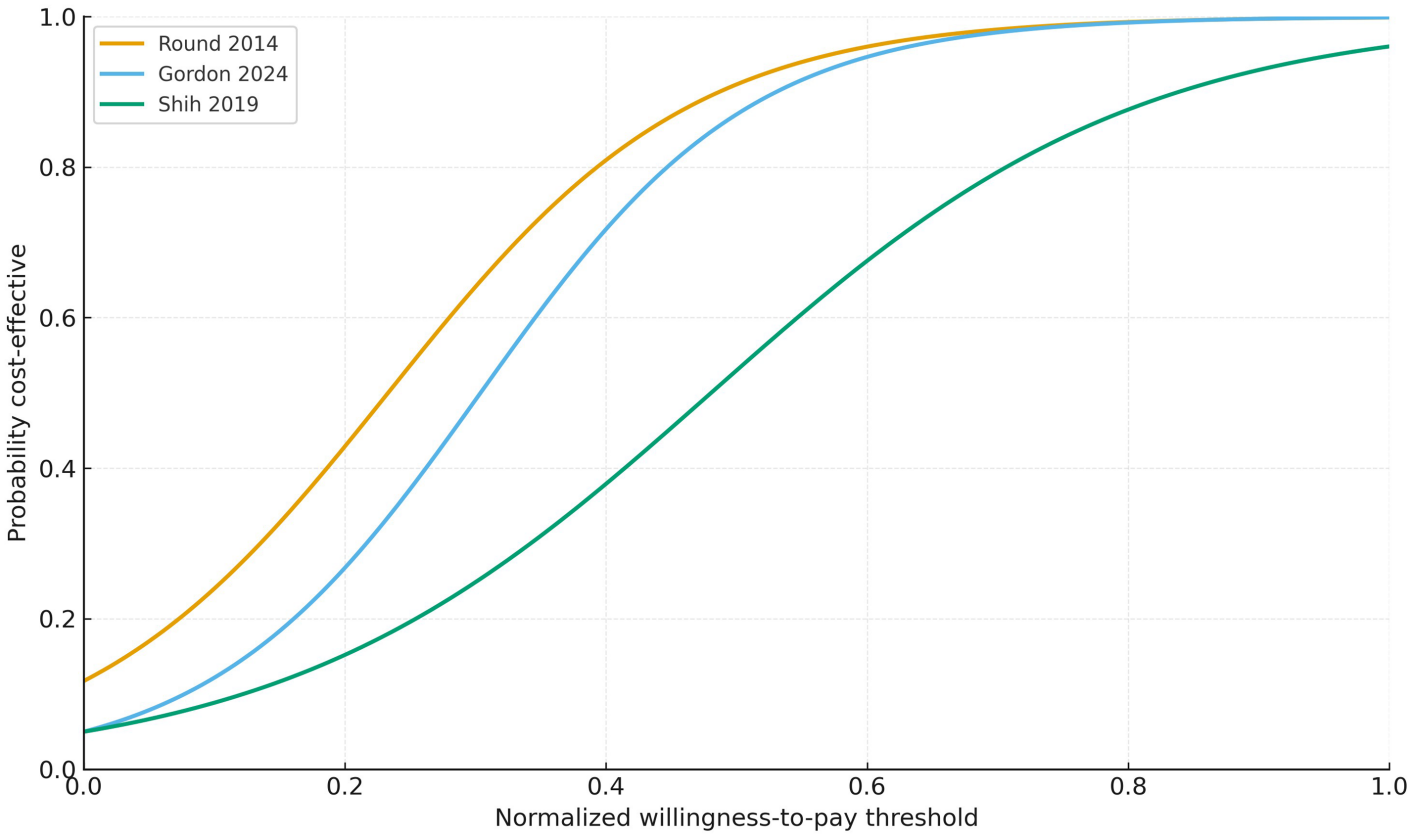
Cost-effectiveness acceptability curves (CEACs) for rehabilitative and psychological integrative oncology interventions reporting probabilistic analyses (30, 32, 33). Curves show the probability of cost-effectiveness across normalised willingness-to-pay thresholds (0 = lowest, 1 = highest). Orange curve, rehabilitative IO modality (Round et al.); blue curve, psychological IO modality (Gordon et al.); green curve, psychological IO modality (Shih et al.).

Gordon et al., representing a psychological IO intervention, showed similarly favourable but slightly lower probabilities, with a steeper increase at intermediate WTP levels (32), see blue curve in Figure 4. Shih et al., also evaluating a psychological IO intervention, displayed the lowest and slowest-rising CEAC, indicating greater uncertainty and a reduced probability of cost-effectiveness at lower WTP thresholds, see green curve in Figure 4 (33). Overall, the CEACs highlight meaningful differences in economic robustness across interventions, while collectively supporting favourable or dominant cost-effectiveness.

## Summary of main findings

This systematic review synthesised 10 CEAs of integrative oncology interventions. Included studies spanned diverse geographic and clinical contexts and addressed a broad spectrum of cancer types, most frequently breast cancer, followed by lung, ovarian, pancreatic, and mixed malignancies. Across study designs, including six RCTs, three RWD studies, and one model-based simulation, the magnitude of economic benefit was moderate yet consistent. IO interventions generally either achieved cost savings or yielded ICERs well below accepted WTP thresholds, alongside measurable improvements in patient-reported outcomes, HRQL or survival. Across studies reporting probabilistic results, the probabilities of cost-effectiveness at national thresholds ranged from approximately 45 to 87%, reflecting variation comparator, time horizon and analytic perspective. No intervention was dominated, and none demonstrated clear cost-ineffectiveness. The CHEERS-based assessment further contextualises these findings. Reporting and costing domains demonstrated strong adherence to methodological standards, supporting confidence in the comparability of economic outcomes. By contrast, sensitivity analysis and especially stakeholder engagement showed lower and more variable performance, largely reflecting the methodological constraints of RWD studies and the rarity of pre-specified health-economic analysis plans. These variations highlight areas where future IO CEAs, particularly RWD-based evaluations, could be strenghtened through enhanced uncertainty analysis, transparency of costing methods, and structured involvement of patients and other stakeholders.

The evaluated interventions comprised phytotherapeutic add-on therapies (*Viscum album L.*, traditional Chinese herbal medicine), psychological programmes (including multimodal psychological support), rehabilitative therapies (including multimodal thermal treatment with physical activity and dietetic consultation), and mind–body techniques (such as acupressure). Across all intervention categories, IO modalities demonstrated favourable or dominant cost-effectiveness profiles. Eight of the 10 studies reported ICERs below their respective national WTP thresholds (13, 16, 27, 30, 31, 33, 34), while three interventions were dominant (28, 29, 32), achieving improved health outcomes at lower or comparable costs. Phytotherapeutic and mind–body interventions exhibited the strongest economic performance, reflected by the lowest relative ICER values (0.3 and 0.4, respectively) (13, 16, 27, 31). Rehabilitative and psychological programmes also showed favourable CEA with relative ICER values around 0.5 and absolute ICERs well below national WTP thresholds (30, 33, 34), while relative ICER values need to be interpreted cautiously due to their exploratory nature. Probabilistic sensitivity analyses likewise supported the stability of these findings, yet these results should be viewed with caution given the small number of studies. The rehabilitative intervention demonstrated the highest economic robustness, with rapidly rising probabilities of cost-effectiveness across WTP thresholds (30). Psychological interventions showed intermediate to lower probabilities, reflecting meaningful variation in economic performance across modalities, while remaining broadly within acceptable cost-effectiveness ranges (32, 33). Taken together, these findings indicate that integrative approaches in oncology consistently provide high-value, patient-centred cancer care across tumour entities, economic perspectives and health system settings by improving HRQL and maintaining or extending survival at acceptable or reduced costs.

## Discussion

This systematic review provides consistent evidence that integrative oncology interventions deliver clinically meaningful and economically efficient outcomes across multiple cancer entities and healthcare settings. Within methodological heterogeneity, including RCTs, model-based simulations, and RWD analyses, the direction of results was consistent. IO interventions improved survival or QALYs while maintaining or reducing total costs. Across studies, all intervention types fell well below commonly referenced WTP, revealing that diverse IO modalities offer high-value care across clinical contexts.

Despite the consistency of cost-effectiveness results, methodological quality varied across CHEERS domains. While reporting and costing quality were high with mean scores of greater 88%, sensitivity analyses and stakeholder engagement were less consistently addressed, indicating opportunities for strengthening transparency and participatory research practices in future IO economic evaluations. The observed variability in reporting detail, particularly regarding discounting and price-year adjustments, likely reflects the practical challenges of working with secondary or routine data and heterogeneous cost structures, rather than systematic weaknesses in study quality. As seen internationally, many CEAs based on RWD data are conducted *post hoc* and therefore lack pre-specified health economic analysis plans, which naturally limits reporting completeness (35, 36). While RCTs provide the highest internal validity and minimise confounding through randomisation, their short time horizons and highly selected patient populations may restrict generalisability (37). Conversely, RWD studies capture broader clinical populations and longer-term outcomes, offering important insights into real-world effectiveness and resource use (38, 39). The convergence of findings across both RCTs and RWD in our study therefore strengthens confidence in the underlying economic signal, while underscoring the need for future prospective, multi-centre evaluations that combine the internal validity of RCTs with the external validity of RWD, extend time horizons, integrate equity-sensitive modelling, and systematically engage stakeholders in defining relevant outcomes and perspectives.

Phytotherapeutic interventions, such as add-on *Viscum album* L. therapy and traditional Chinese herbal medicine, were cost-effective in long-term RWD cohorts (13, 16, 27). German RWD analyses further confirmed that phytotherapeutic add-on therapies were associated with fewer hospitalisations, maintained quality of life, and improved patient-reported and clinical outcomes (13, 16, 40). Mind–body interventions, such as acupressure for chemotherapy-induced nausea, reduced adverse effects and improved QALYs at minimal cost (31). Rehabilitative interventions were either dominant, demonstrating lower total costs with improved survival or cost-effective within accepted WTP ranges, with relative ICER values comparable to psychological interventions (22, 28–30). However, these findings on relative ICER values should be interpreted cautiously given the exploratory normalisation approach and the heterogeneity and small number of studies. Rehabilitative programmes improved physical and psychosocial recovery as well as return-to-work outcomes, aligning with international survivorship and palliative care objectives (12, 41). Probabilistic sensitivity analyses for the rehabilitative intervention revealed the highest economic certainty, followed by psychological interventions exhibiting intermediate and lower probabilities respectively, highlighting meaningful variation in economic performance.

Psychological programmes were consistently cost-effective or dominant, yielding measurable improvements in HRQL, emotional well-being, and coping capacity (32–34). Structured counselling and digital psychoeducation interventions effectively reduced fatigue, anxiety, and distress, enhanced self-efficacy, and supported continuity of care during the transition from active treatment to survivorship. Evidence from these psychological RCTs further shows that low-intensity, scalable digital interventions can sustain psychosocial benefit while simultaneously reducing delivery costs. This is an important consideration for equitable cancer care in resource-limited health systems and during periods of disrupted service provision, aligning with the WHO Global traditional medicine strategy’s pillar of accessible models of integrated care. Notably, several CEAs were conducted during or post-COVID-19 pandemic, a period that accelerated digital transformation across psycho-oncology and other supportive care domains while reducing reliance on physical infrastructure and personnel (19, 42). Although pandemic-related restrictions occasionally limited completeness of data collection, the economic efficiency of digital counselling interventions remained stable, underscoring their long-term sustainability, scalability, and relevance for future IO implementation. Within the broader field of integrative medicine, the most comprehensive review to date identified 204 economic evaluations of integrative medicine interventions across diverse clinical conditions. Although many interventions appeared cost-effective, substantial methodological heterogeneity and limited reporting quality constrained the interpretability of findings (5). Importantly, unlike our review, which was specifically designed to capture cancer-related evidence, the search strategy did not include any oncology-specific terms. Consequently, cancer-related evaluations were neither systematically identified or analysed.

In IO, the existing literature is similarly limited. Jatoi et al. reviewed complementary and integrative care among cancer patients but focused primarily on utilisation patterns and patient expenditures rather than extracting formal cost-effectiveness metrics such as ICERs (8). Compared with their work, our review systematically identifies full economic analyses within oncology populations treated with integrative therapies. More recently, Mao et al. provided a valuable global overview of integrative oncology but likewise did not include formal economic comparisons (43). Together, these earlier works highlight the persistent gap in methodologically sound, IO-specific economic evaluations needed to inform reimbursement and policy decisions. Furthermore, a conceptual analysis argues that integrative oncology programmes often operate within structurally problematic economic conditions, characterised by limited reimbursement, fragmented funding pathways and substantial out-of-pocket spending by patients (9). They emphasise that the field of IO lacks robust health-economic evaluation frameworks, making it difficult to demonstrate value within conventional oncology financing models. Our systematic review directly addresses these structural and methodological limitations by systematically identifying all published cost-effectiveness analyses of integrative approaches in oncology, extracting formal ICERs, QALYs, LYG, and probabilistic evidence. In doing so, it may provide, for the first time, a consolidated and methodologically sound economic evidence base in IO.

Overall, this systematic review shows that integrative oncology may provide not only meaningful clinical benefits but also favourable economic value, aligning patient-centred outcomes with the broader goals of sustainable and efficient health-system performance. In the context of growing international commitment, exemplified by the WHO Global traditional medicine strategy 2025–2034, these findings underscore a timely opportunity to advance evidence-informed policy, expand equitable access, and integrate cost-effective IO approaches into routine cancer care worldwide.

### Strengths and limitations

A limitation of this review is that several included studies reported incomplete sensitivity analyses, limited stakeholder engagement, and no assessment of equity or distributional effects (5, 44). Further, although the likelihood is low, it remains possible that some studies of single IO interventions were not identified, as no globally standardised nomenclature for integrative medicine interventions currently exists. Most RCT-based evaluations used short analytic horizons, potentially underestimating long-term benefits and downstream costs, and thus likely suggesting lower cost-effectiveness than may be the case in the longer term (12, 45). Conversely, early trial-based improvements could overstate value if maintenance costs or delayed adverse events are not fully incorporated (12). Cost components, valuation methods, and currency conversions varied across studies, limiting comparability. The predominance of single-centre or national studies may restrict generalizability and transferability and lead to mis-estimation of economic value applied to different health-system contexts (46). Moreover, equity-related factors—such as socioeconomic or insurance-based differences—were rarely examined, and the limited use of equity-focused and distributional cost-effectiveness analyses leaves uncertainty about differential access, uptake, and benefits across socioeconomic or insurance groups (10, 47). Likewise, insufficient attention to affordability across health systems and limited stakeholder engagement in defining relevant outcomes and perspectives may have constrained the scope and policy relevance of existing evaluations (48). Addressing these gaps would refine cost-effectiveness estimates and better align future research with priorities on equity, access, and person-centred cancer care (15). The systematic review reflects the state of the evidence up to April 2025, and although a targeted check of recent publications was performed, relevant studies meeting the inclusion criteria and published after this date may not have been comprehensively captured. Although no language restrictions were applied, the search relied primarily on English-language databases. Consequently, some non-English publications may have been missed if they were not indexed with English titles or abstracts. Publication bias cannot be excluded, as unfavourable or non–cost-effective findings are less likely to appear in the literature. Cross-national transferability of economic results is inherently limited. Marked international variation in resource use, unit costs, epidemiology, and reimbursement systems cannot be fully reconciled through standard costing adjustments, limiting transferability across jurisdictions (49), these adaptations were beyond the scope of our review. Since CHEERS primarily assesses reporting quality and not study design, differences between RCTs, model-based analyses, and RWD studies may not be fully reflected. Common limitations across the evidence base included small sample sizes, single-centre designs, and the short time horizons typical of RCTs—contrasting with the longer follow-up or life-long modelling found in RWD and model-based studies. While RCTs enrol narrowly defined patient populations and RWD studies may be subject to selection bias and residual confounding, both designs face challenges in generalising findings across different health-system contexts. In RWD analyses, residual confounding may either inflate or attenuate cost-effectiveness by attributing observed differences in outcomes or costs to the intervention when they may partly reflect unmeasured patient characteristics, underlying disease severity, or differences in care pathways (50). Despite these constraints, most analyses achieved high CHEERS reporting scores, and reviewer agreement was strong. A major strength of this review is its comprehensive synthesis of all published economic evaluations of IO interventions, spanning diverse cancer types, modalities, and perspectives. The inclusion of trial-based, model-based, and real-world evidence enhances external validity. The combined use of CHEERS appraisal, probabilistic interpretation, and alignment with WHO policy frameworks may provides a methodologically robust foundation for future IO economic evaluations.

## Conclusion

Taken together, the findings of this systematic review suggest that integrative oncology interventions evaluated in our analyses contribute to value-based cancer care by improving clinical and patient-reported outcomes without increasing health-system expenditure; however, given that cost-effectiveness evidence is not available for all modalities, these conclusions should be subject to further confirmation. Our analyses reveal that phytotherapeutic, psychological, mind–body, and rehabilitative interventions consistently showed favourable or dominant cost-effectiveness. Among these, phytotherapeutic and mind–body interventions showed the most favourable relative ICER values, indicating the strongest cost-effectiveness in absolute terms, although this should be interpreted cautiously given the exploratory normalisation and underlying heterogeneity. Rehabilitative programmes exhibited the highest probabilistic robustness, with the steepest and highest cost-effectiveness acceptability curves across willingness-to-pay thresholds.

To our knowledge, this review represents the first systematic synthesis of cost-effectiveness evidence of integrative oncology explicitly framed within the WHO Global traditional medicine Strategy 2025–2034, thereby providing a consolidated economic perspective to support policy relevant decision-making.

The findings are broadly consistent with the WHO Strategy’s priorities of strengthening evidence generation, promoting equitable access to whole person care, and integrating safe and effective complementary practices into national cancer-control frameworks. From a policy perspective, these results suggest that healthcare systems should consider increasing access to integrative oncology treatment option and exploring the integration of traditional and complementary interventions into cancer care. In addition, health insurances may consider reimbursement for rehabilitative, phytotherapeutic, mind–body and psycho-oncological treatments when clear patient-relevant and system-level benefits have been demonstrated. Future research on the costs and benefits of integrative oncology should be further promoted and adequately funded. Such research should involve multi-centre designs, systematically capture patient experiences and outcomes, apply standardised cost-measurement approaches, and assess fairness. This would improve the quality of the evidence and its relevance for policy decision-making. In summary, the available economic evidence suggests that integrative oncology has the potential to help create cancer care that better supports patients, uses financial resources efficiently, and promotes fairness, thereby strengthening cancer care systems for the future. These implications should be considered in the context of the current evidence base and the need for further research.

## Data Availability

The original contributions presented in the study are included in the article/Supplementary material, further inquiries can be directed to the corresponding authors.

## Glossary

WHO: World Health Organisation
RCT: randomised controlled trial
IO: integrative oncology
TCIM: Traditional, Complementary, and Integrative Medicine
TCIH: Traditional, Complementary, and Integrative Healthcare
PROSPERO: International Prospective Register of Systematic Reviews
PRISMA-P: Preferred Reporting Items for Systematic Review and Meta-Analysis Protocols
CCCTIM: Charité Competence Center for Traditional and Integrative Medicine
FIH: Research Institute Havelhöhe
IVAA: International Federation of Anthroposophic Medical Associations
PICO: Population, Intervention, Comparison, Outcome
CINAHL: Cumulative Index to Nursing and Allied Health Literature
ISPOR: International Society for Pharmacoeconomics and Outcomes Research
CHEERS: Consolidated Health Economic Evaluation Reporting Standards
ICER: incremental cost-effectiveness ratio
WTP: willingness-to-pay
PSA: probabilistic sensitivity analysis
CEAC: cost-effectiveness acceptability curve
HRQL: health-related quality of life
RWD: real-world data
LYG: life-year gained
PPP: purchasing-power parity
OOP: out-of-pocket costs
