# Supplementary material for: Cost-effectiveness of integrative oncology for sustainable and patient-centred cancer care: a systematic review in the context of the WHO global traditional medicine strategy 2025–2034
Source: Front Public Health. 2026 Mar 18;14:1773455. doi: 10.3389/fpubh.2026.1773455 (PMC13038974; doi:10.3389/fpubh.2026.1773455)
Supplement: Supplementary file 1 [file Data_Sheet_1.pdf]

Supplementary figures and tables

Supplementary table S1. PRISMA-P 2015 Checklist

Topic	Page number(s)
Title: Identification (1a)	p. 1
Title: Update (1b)	—
Registration (2)	p. 2
Authors: Contact (3a)	p. 1
Authors: Contributions (3b)	p. 23
Amendments (4)	—
Support: Sources (5a)	p. 23
Support: Sponsor (5b)	p. 23
Support: Role of sponsor/funder (5c)	p. 23
Introduction: Rationale (6)	p. 3–4
Introduction: Objectives (7)	p. 3
Methods: Eligibility criteria (8)	p. 4
Methods: Information sources (9)	p. 5
Methods: Search strategy (10)	p. 5 and supplementary source
Study records: Data management (11a)	p. 5
Study records: Selection process (11b)	p. 5
Study records: Data collection process (11c)	p. 5
Data items (12)	p. 5
Outcomes and prioritization (13)	p. 5-6
Risk of bias in individual studies (14)	p. 6
Data: Synthesis (15a)	p. 7
Data: Synthesis (15b)	—
Data: Synthesis (15c)	p. 7
Data: Synthesis (15d)	p. 7
Meta-bias(es) (16)	—
Confidence in cumulative evidence (17)	p. 7

PRISMA-P 2015 Checklist, adapted for the use with protocol submissions to Systematic Reviews from Moher et al. (36)

Supplementary table S2. Search strategy

Boolean search strategy; advanced search 08.04.2025		
1	*Integrative OR integrated OR complementary OR alternative OR holistic OR traditional OR whole person OR whole system OR non-pharmacol* OR supportive OR herb* OR plant* OR mistletoe OR add-on OR concomitant OR viscum OR supplement* OR ayurved* OR chines* OR anthropos* OR TCM OR Asian OR oriental OR homeopath* OR globul* OR taiji OR tai ji OR tai-chi OR tai chi OR qigong OR qi gong OR chikung OR chi kung OR exercise OR physical OR mind-body OR relax* OR breathing OR mindful OR rehabil* OR yangsheng OR yang sheng OR nurs* OR counselling OR counseling OR electro* OR supervision OR nutrition OR music OR art OR yoga OR behavi* OR mental OR psycho* OR spiritual OR vitamin OR botanic* OR phyto* OR ginger OR garlic OR neem OR green tea OR turmeric OR berberine OR astragalus OR ginseng OR mushroom OR Echinacea OR manuka honey OR clove OR acupunct* OR acupress* OR neural therap* OR dry needl* OR stress OR anxiety OR distress OR fatigue OR pain OR hypno* OR palliat* OR depression OR nausea OR xerostomia OR healing OR bioenergy OR reiki therapy OR touch OR helleborus OR quality of life OR massage OR eurythm* OR surviv* OR remission OR recovery OR efficacy OR safety OR efficiency*	4,775,163 [1]
2	*cost-eff* OR cost eff* OR cost of illness OR cost benefit analysis OR cost utility analysis OR health care cost OR finance* OR health care financing OR economic evaluation OR economic aspect OR economic*<br>*health economics OR cost minimization analysis OR direct cost*<br>*indirect cost OR out-of-pocket cost OR health expenditure OR budget impact analysis OR financial burden OR reimbursement OR payment model OR insurance coverage OR cost saving OR value-based care*<br>*return on investment OR economic impact OR pharmacoeconomic*<br>*economic modeling OR health economic model OR resource allocation*<br>*cost allocation OR economic outcome*	68,015[1]
3	*onco* OR cancer* OR neoplas* OR metasta* OR carcino* OR malign*<br>tumor* OR tumour* OR lymphom* OR leukem* OR sarcom**	4,169,440[1]
4	1 AND 2 AND 3	7,955

[1]the numbers represent the absolute number of hits obtained from the database for search line #1, #2, and #3 before applying any Boolean combinations or filters in #4

Supplementary table S3. Diagnosis based subgrouping of CEA analyses

Diagnosis, indication	Studies and study design
Breast cancer (n=4)	Mandelblatt 2008 RCT (17)
	Round 2014 RCT (13)
	Mourgues 2014 RCT (12)
	Shih 2019 RCT (16)
Non-small cell lung cancer (n=2)	Thronicke 2020b RWD (9)
	Tang 2024 RWD (10)
Pancreatic cancer (n=1)	Thronicke 2020a RWD (8)
Ovarian cancer (n=1)	Dholakia 2021 model (11)
Brain tumors (n=1)	Gordon 2024 RCT (15)
Mixed or multiple cancers (n=2)	Molassiotis 2013 RCT (14)
	Shih 2019 RCT-(14)

RCT, randomized controlled trial; RWD, real-world data; model, model-based; n, number

Supplementary table S4. Subgrouping of included CEA-analyses by economic perspective

Economic perspective	Studies and study design
Hospital or provider perspective (n=3)	Thronicke 2020a RWD (8)
	Thronicke 2020b RWD (9)
	Mourgues 2014 RCT (12)
Health-care or payer perspective (n=5)	Dholakia 2021 model (11)
	Gordon 2024 RCT (15)
	Tang 2024 RWD (10)
	Molassiotis 2013 RCT (14)
	Round 2014 RCT(13)
Societal perspective (n=2)	Mandelblatt 2008 RCT (17)
	Shih 2019 RCT (16)
Mixed or multi-perspective analyses (n=1)	Molassiotis 2013 RCT (14)

RCT, randomized controlled trial; RWD, real-world data; model, model-based; n, number

Supplementary table S5. Subgrouping of included CEA-analyses by intervention category

Intervention category	Studies and study design
Phytotherapy (n=3)	Thronicke 2020a RWD (8)
	Thronicke 2020b RWD (9)
	Tang 2024 RWD (10)
Psychological support (n=3)	Mandelblatt 2008 RCT (17)
	Gordon 2024 RCT (15)
	Shih 2019 RCT (16)
Rehabilitative programs (n=3)	Mourgues 2014 RCT (12)
	Round 2014 RCT (13)
	Dholakia 2021 model (11)
Mind-body therapy (n=1)	Molassiotis 2013 RCT (14)

RCT, randomized controlled trial; RWD, real-world data; model, model-based; n, numbee

Supplementary table S6. Relative ICER scores across integrative oncology intervention categories

Study	Relative ICER score	Relative ICER score[1]	Mean relative ICER score
**Phytotherapy**			**0.3**
Thronicke 2020a	€7,539/€50,000=0.15	0.2	
Thronicke 2020b	€3,586/€50,000=0.07	0.1	
Tang 2024	*NT$*880,908/ *NT$*1,792,062=0.49	0.5	
**Psychological**			**0.5**
Mandelblatt 2008	€7,275/€50,000=0.15	0.2	
Shih 2019	AU$34,300/ AU$50,000=0.69	0.7	
**Rehabilitativ**			**0.5**
Round 2014	£14,231/£30,000=0.47	0.5	
**Mind-body**			**0.4**
Molassiotis 2013	7,360/20,000= 0.37	0.4	

Normalized incremental cost-effectiveness ratios (ICERs) by intervention category, expressed relative to national willingness-to-pay thresholds; [1]rounded; Relative ICERs were calculated by dividing each study's reported ICER by the respective country-specific WTP benchmark
